# Bu Yang Huan Wu Prevents Osteogenic Effect of Muscle‐Derived Stromal Cells via Regulating JAK/STAT Pathway

**DOI:** 10.1111/jcmm.70413

**Published:** 2025-02-12

**Authors:** Guorui Cao, Shaoyun Zhang, Yuanping Liao, Chen Yue, Lanbo Yang, Jiayi Guo, Peijian Tong, Honglue Tan

**Affiliations:** ^1^ Department of Knee Surgery Luoyang Orthopedic Hospital of Henan Province, Orthopedic Hospital of Henan Province Luoyang Henan Province People's Republic of China; ^2^ Department of Orthopedics, The Third Hospital of Mianyang Sichuan Mental Health Center Mianyang Sichuan Province People's Republic of China; ^3^ Hunan University of Chinese Medicine Changsha Hunan Province People's Republic of China; ^4^ The First Affiliated Hospital of Zhejiang Chinese Medical University Hangzhou Zhejiang Province People's Republic of China

**Keywords:** Bu Yang Huan Wu, macrophage, muscle‐derived stromal cells, oncostatin M, the janus kinase/signal transducer and activator of transcription

## Abstract

Heterotopic ossification (HO) is a crucial pathological process in which bone or calcification develop in skeletal muscle and surrounding soft tissues. Muscle‐derived stromal cells (MDSC) are important muscle‐resident mesenchymal progenitor cells and macrophage‐derived oncostatin M (OSM) can induce osteogenic differentiation. Bu Yang Huan Wu (BYHW), which has a long history of use in restraining inflammation, can prevent osteogenic differentiation and HO formation while underlying mechanism is still unclear. The Janus kinase/signal transducer and activator of transcription (JAK/STAT) signalling pathway is an important pathway to regulate osteogenic differentiation of related cells. In this study, we investigated whether BYHW could inhibit osteogenesis effect of MDSC through OSM mediated by macrophages, and whether JAK/STAT pathway regulated this biological process. We found that activated macrophages promoted osteogenic differentiation of MDSC through OSM and BYHW could decrease the level of OSM and osteogenic activity of MDSC. Further, we confirmed the regulatory effect of JAK/STAT pathway, blocking this pathway could suppress the level of OSM and osteogenic differentiation of MDSC. We showed that BYHW could suppress osteogenic differentiation of MDSC through JAK/STAT signalling. These findings expand the application scope of traditional Chinese medicine and provide a basis for the further investigation of the potential therapeutic role of HO.

## 1 Introduction

Acquired heterotopic ossification (HO) is the process of bone formation or calcification in non‐skeletal tissues, which could be triggered by fracture, burn, combat‐related injuries, arthroplasty and nervous system injuries [1, 2, 3]. This condition may lead to pain, joint stiffness and the loss in quality of life [4]. Current HO treatment options mainly included radiation therapy, nonsteroidal anti‐inflammatory drugs and surgical removal. Because of the cognition gap of pathogenesis, related treatment is always inefficient and nonspecific, even associated with risk of recurrence [5].

The formation of HO requires the participation of osteogenic progenitor cells and appropriate osteogenic microenvironment. Muscle‐derived stromal cells (MDSC) are muscle resident mesenchymal progenitor cells that have osteogenic differentiation potential. As reported, abnormal osteogenic differentiation and enhanced osteogenic activity of MDSC are the essential reasons of HO [6, 7, 8]. In addition, related evidence suggests that inflammation is the main trigger of osteogenic differentiation and acquired HO [9, 10]. It has been reported that macrophage‐derived oncostatin M (OSM) is a key mediator of HO, which could induce osteogenic differentiation of related cells and promote matrix mineralisation [11, 12, 13]. The authors demonstrated that macrophages contributed to HO formation through the osteogenic action of OSM on muscle cells within an inflammatory context and OSM/OSM receptor could be a suitable therapeutic target [13]. The evidences above show that OSM secreted by macrophages could induce osteogenic differentiation of MDSC, eventfully leads to acquired HO.

As a classic traditional Chinese medicine (TCM), Bu Yang Huan Wu (BYHW) decoction has the effects of qi‐tonifying and stasis‐eliminating. Modern medical researches indicate BYHW can inhibit inflammation and the activation of macrophages, suppress osteogenic differentiation of stem cells and prevent the incidence of HO [14, 15, 16, 17]. However, the mechanism underlying BYHW against osteogenic differentiation of stem cells and HO formation remains unclear. The janus kinase‐signal transducer and activator of transcription (JAK/STAT) signalling pathway is a cytokine‐stimulated signal transduction pathway and involved in various biological processes, including differentiation, immune regulation and proliferation [18]. Recent studies showed that macrophages could secrete OSM cytokines, which promoted osteogenic differentiation of ligamentum flavum cells mainly through the JAK/STAT signalling pathway. Inhibition of JAK1/2 tyrosine kinases could reduce the incidence of HO [19, 20]. Moreover, BYHW has been shown to inhibit JAK/STAT pathway [21].

In this study, we firstly asked whether BYHW could inhibit the level of OSM and osteogenic activity of MDSC. In addition, due to the critical role of JAK/STAT signalling in the pathogenesis of cell osteogenic differentiation and HO formation, we investigated whether BYHW mitigates the level of OSM secreted by macrophages against osteogenic differentiation of MDSC through regulating JAK/STAT signalling.

## 2 Materials and Methods

### 2.1 Preparation of BYHW Serum

All the herbal constituents of BYHW were obtained from Hunan University of Chinese medicine, with drug lot numbers as Huang Qi (CK23101701), Dang Gui (TH23092201), Di Long (20220927), Chi Shao (SN23091802), Chuan Qiong (2309072), Tao Ren (221201) and Hong Hua (2023080808). The BYHW composition portion was set according to the dose originally recorded in Yilin Gaicuo.

Adult male Sprague–Dawley rats (250 ± 20 g) were purchased from Hunan SJA Laboratory Co. Ltd. (Changsha, Hunan, China). The experimental animal production licence was SYXK (Xiang) 2019–0009. After 1 week of adaptive feeding, the rats were randomly divided into negative control group (*n* = 5) and BYHW group (*n* = 10). The BYHW group was fed 2 mL of BYHW decoction two times every day and the perfusion continued for 1 week. The negative control group was fed with an equal volume of distilled water. The blood was taken from abdominal aorta, and then centrifuged at 3000 r/min. The supernatant was filtered, sterilised and packed, then stored in refrigerator at −20°C for later use.

### 2.2 Preparation of MDSCs and Macrophages

MDSCs were a gift from Institute of Sports Medicine of Peking University. They were cultured in Dulbecco's Modified Eagle Medium (DMEM, abiowell#AW‐M003, China) supplemented with 10% foetal bovine serum (FBS, abiowell#AWC0219a, China), 100 U/mL penicillin‐100 μg/mL streptomycin solution (abiowell#AWH0529, China). RAW264.7 macrophages were purchased from Chinese Academy of Sciences (#TCM13, China). Cultures were maintained in a humidity incubator (Santn#DH‐160I, China) with 5% CO_2_ at 37°C. Culture medium (Abiowell#AW‐MC037, China) was changed every 2–3 days. When the cells were grown to 80% confluence, they were transferred to Transwell Permeable Supports, Polycarbonate Membrane (Corning#3412, USA). The upper chamber was macrophage and lower was MDSC. The cells were treated with blank serum, BYHW drug‐contained serum and short hairpin RNA against JAK (HonorGene#HG‐LV146145sh1/sh2/sh3 and #HG‐LV008413sh1/sh2/sh3, China) for subsequent experiments.

### 2.3 JAK Knock‐Down Lentiviral Transduction

Lentiviruses encoding mouse JAK1 and JAK2 short hairpin RNA (LV‐shJAK1 and LV‐shJAK2) or control shRNA (LV‐shcontrol) were purchased from HonorGene (Changsha, China). The virus titre was 2.0 × 10^8^, the multiplicity of infection value of MDSC was 50, and the number of cells was 1.0 × 10^5^. Virus volume = (cell number × multiplicity of infection)/virus titre. The co‐cultured cells were incubated with transfection aid reagent and DMEM for 72 h.

### 2.4 Cell Culture and Experimental Protocols

To determine how BYDW would affect osteogenesis‐promoting effect of MDSC through OSM mediated by macrophages, the MDSC and macrophages were co‐cultured (the ratio of MDSC and macrophages was 1:10), then divided into seven groups on the basis of different interventions: blank serum group, 10% BYHW (serum was added to lower chamber), 20% BYHW, sh‐JAK1 (lentiviruses were added to lower chamber), sh‐JAK2, 20% BYHW+sh‐JAK1 (serum and lentiviruses were added to lower chamber) and 20% BYHW+ sh‐JAK2. The drug intervention was usually started on the third day of co‐culture. After co‐culture of 14 days, we collected cell or lysates sample for further analysis. The level of OSM and osteogenic protein was determined using a commercial enzyme‐linked immunosorbent assay (ELISA) kit (CUSABIO, Wuhan, China) and Western blotting (WB). Related gene expression was determined by quantitative real‐time reverse‐transcription polymerase chain reaction (qRT‐PCR) analysis (see below).

### 2.5 Histology

The cells were fixed with 4% paraformaldehyde (Abiowel#AWI0056, China) for 30 min at room temperature and then washed two times using PBS (Abiowell#AWC0214a, China). For Alizarin Red staining, the cells were soaked in Alizarin red dye solution for 3 min. Immunohistochemistry was performed according to protocols described for the alkaline phosphatase (ALP) staining kit (Abiowell#AWI0314a, China). Pictures were captured using an inverted microscope (Cnmicro, Beijing, China).

### 2.6 qRT‐PCR Analysis

Basing on manufacturer's instructions (CWBIO, Beijing, China), cells were lysed in QIAzol Lysis Reagent and isolated using the RNeasy Mini Kit. RNA was converted to complementary DNA using the SuperScript IV First‐Strand Synthesis System, and levels of expression of related genes were quantitated with real‐time PCR using the SYBR Green PCR Master Mix (ABI‐invitrogen, USA). Gene expression level was calculated using the 2‐△△Ct method. Primer sequences for actin, ALP, osteocalcin (OCN), runtrelated transcription factor 2 (Runx2), OSM, STAT1, STAT3, JAK1 and JAK2 are listed in Table 1.

**TABLE 1:** PCR primer sequences.

Genes	Forward	Reverse
Actin	CTCCTGAGCGCAAGTACTCT	TACTCCTGCTTGCTGATCCAC
ALP	GGGCCTGCTCTGTTTCTTCA	CTGAGATTCGTCCCTCGCTG
OCN	GAACAGACAAGTCCCACACAGC	TCAGCAGAGTGAGCAGAAAGAT
RUNX 2	ACTCCAAGACCCTAAGAAACCGAT	TGGCTCCTCCCTTCTCAACCTC
OSM	AATTTCTGAAGACTCCGGCTTT	GGCCATGCAGAAAACATTGTTCC
STAT1	AGTCAGGGCAAGACATCCACT	CCACTTTAAGCTCTGCCGCCTCA
STAT3	CAATACCATTGACCTGCCGAT	GAGCGACTCAAACTGCCCT
JAK1	GAGCTGCACCGACTTTGACAAC	GTAGCGGCCCTTCTGTACCTCA
JAK2	ACTGGACTATATGTGCTACGA	GTTCCTCTTAGTCCCGCTGA

Abbreviation: PCR, polymerase chain reaction.

### 2.7 Western Blotting Analysis

Cells were lysed immediately in RIPA (Abiowel#AWB0136, China) for 10 min at 4°C. The supernatant was transferred to a 1.5 mL centrifuge tube following centrifugation. Protein concentration was determined using the bicinchoninic acid assay (Abiowel#AWB010, China). The mixed solution was analysed by SDS/PAGE (Abiowel#AWT0047, China) and transferred to polyvinylidene difluoride membrane. Primary antibodies directed against β‐actin (proteintech#66009‐1‐Ig, USA), ALP (Affinity#AF12427, Australia), OCN (Affinity#AF12303, Australia), RUNX2 (Affinity#AF5186, Australia), p‐JAK1 (Abiowell#AWA44461, China), p‐JAK2 (Abiowell#AWA41311, China), p‐STAT1 (Abiowell#AWA45521, China) and p‐STAT3 (Abiowell#AWA00673, China) were incubated at 4°C overnight, followed by goat anti‐mouse IgG secondary antibody (Abiowell#AWS0001, China) or goat anti‐rabbit IgG secondary antibody (Abiowell#AWS0002, China) incubation. The band intensity of each blot was quantified by Image J software (JAVA, USA).

### 2.8 Statistical Analysis

All statistical analyses were performed by using SPSS version 25.0 (SPSS Inc. USA). The data were expressed as mean ± standard deviation. Differences among different groups were assessed through one‐way analysis of variance followed by the Tukey multiple comparisons test. A *p*‐value < 0.05 was regarded as statistical significance.

## 3 Results

### 3.1 Activated Macrophages Promoted Osteogenic Differentiation of MDSC Through OSM

**FIGURE 1:**
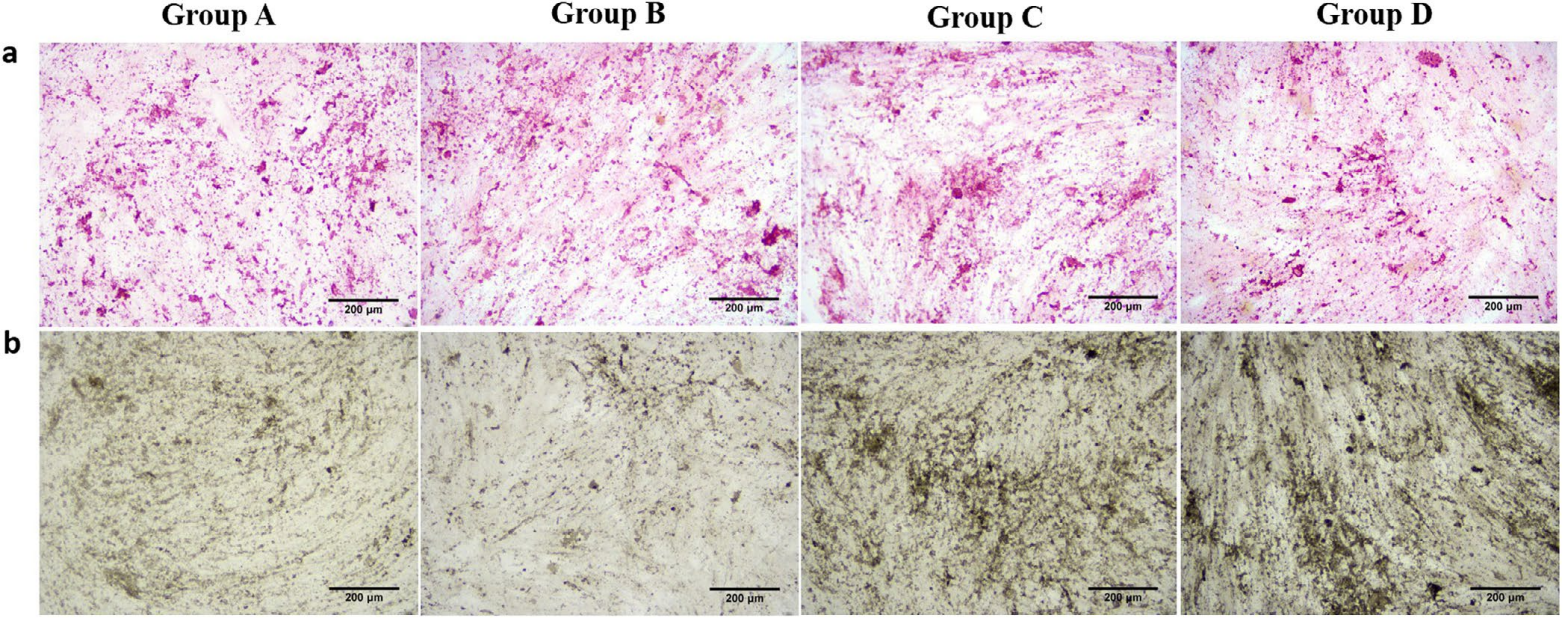
Activated macrophages promoted osteogenic differentiation of MDSC through OSM (Histology). (a) Alizarin Red staining demonstrating calcium deposition in different groups. (b) Immunohistochemistry showing the presence and distribution of ALP in different groups. Group A: MDSC, group B: MDSC+macrophage conditioned medium +OSM neutralising antibody, group C: MDSC+OSM, group D: MDSC+macrophage conditioned medium. Bar = 200um. MDSC = Muscle‐derived stromal cells; ALP = alkaline phosphatase; OSM = oncostatin M.

**FIGURE 2:**
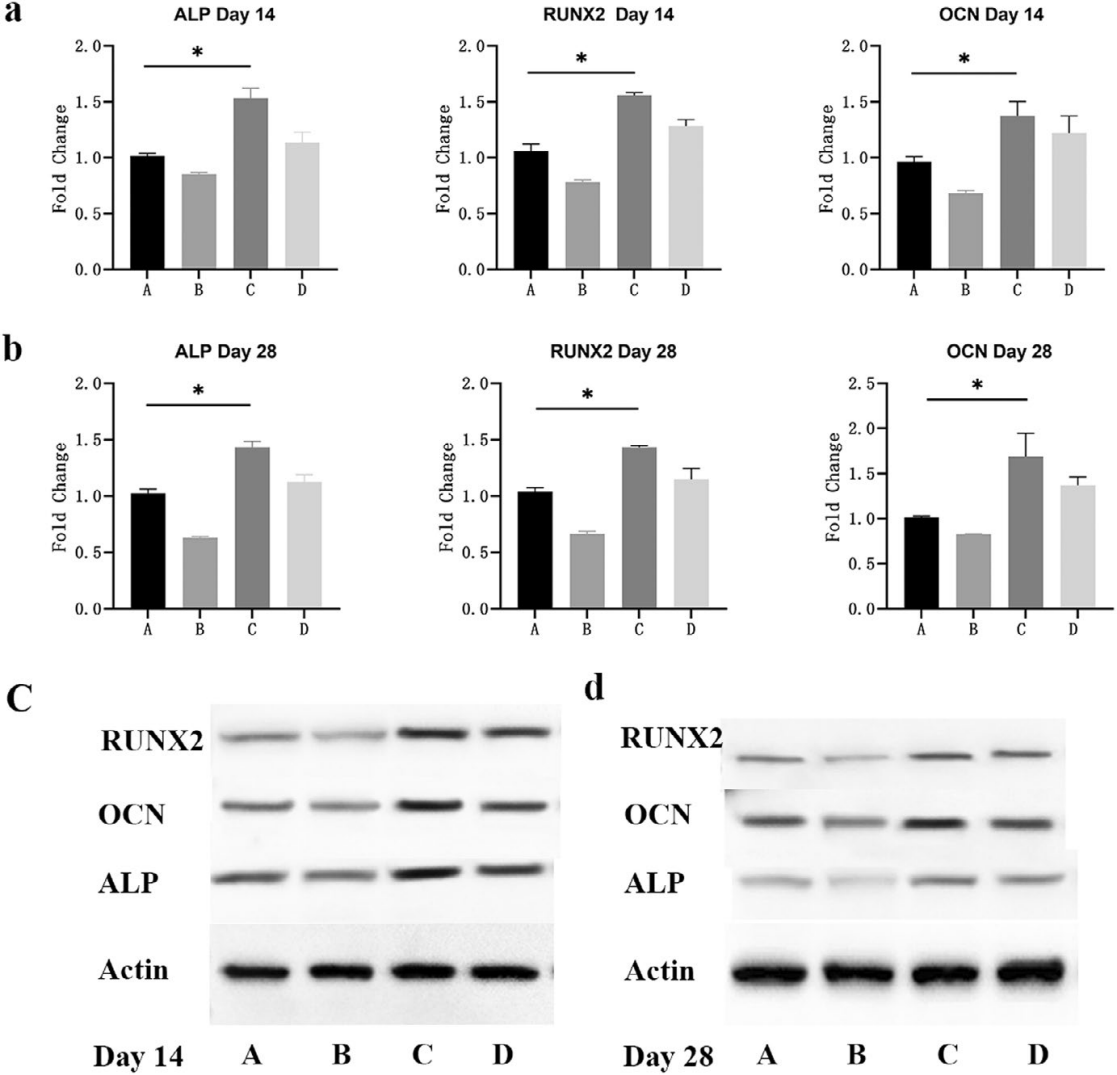
Activated macrophages promoted osteogenic differentiation of MDSC through OSM (PCR and WB). PCR and WB were performed to measure osteogenic gene and protein expression on days 14 and 28 in different groups. (a) PCR analysis of the expression profile of ALP, RUNX2 and OCN on day 14. (b) PCR analysis of the expression profile of ALP, RUNX2 and OCN on day 28. (c) WB analysis of the expression profile of ALP, RUNX2 and OCN on day 14. (d) WB analysis of the expression profile of ALP, RUNX2 and OCN on day 28. Group A: MDSC, group B: MDSC+macrophage conditioned medium +OSM neutralising antibody, group C: MDSC+OSM, group D: MDSC+macrophage conditioned medium. * indicates *p* < 0.05. MDSC = Muscle‐derived stromal cells; PCR = polymerase chain reaction; WB = Western blotting; ALP = alkaline phosphatase; RUNX2 = runtrelated transcription factor 2; OCN = osteocalcin.

To determine the crosstalk of macrophages and MDSC, MDSC was divided into four groups (group A: MDSC, group B: MDSC+macrophage conditioned medium +OSM neutralising antibody, group C: MDSC+OSM, group D: MDSC+macrophage conditioned medium). Two weeks later, Alizarin Red showed calcium deposition increased obliviously in groups C and D compared with groups A and B (Figure 1a). Immunohistochemistry confirmed this finding, showing massive positive staining of ALP in groups C and D (Figure 1b). In addition, we collected MDSC in different groups on day 14 and day 28. PCR results indicated significantly decreased levels of osteogenic gene (ALP, OCN and RUNX2) in groups C and D (Figure 2a,b). Western‐Blot examined similar expression profile of osteogenic protein (ALP, OCN and RUNX2) (Figure 2c,d). The gene and protein expression decreased in group B and increased in groups C and D compared with group A. These indicated that the OSM secreted by macrophages could induce osteogenic differentiation of MDSC.

### 3.2 BYHW Inhibited Osteogenesis‐Promoting Effect of MDSC Through OSM Mediated by Macrophages

**FIGURE 3:**
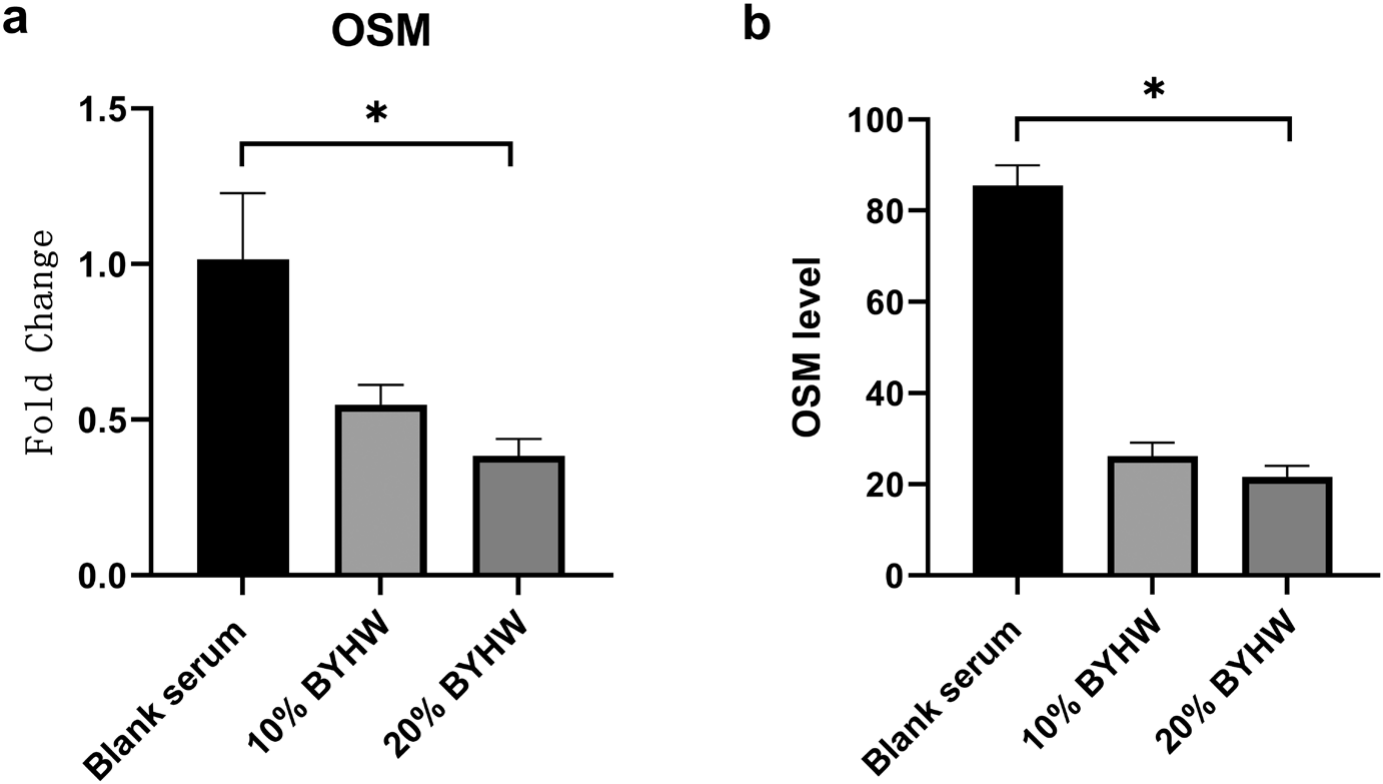
BYHW inhibited the level of OSM. OSM level in co‐cultures cell measured by PCR and ELISA. (a) The expression at the gene level;(b) The expression at the protein level; * indicates *p* < 0.05. BYHW = Bu Yang Huan Wu. OSM = oncostatin M; PCR = polymerase chain reaction; ELISA = enzyme‐linked immunosorbent assay.

**FIGURE 4:**
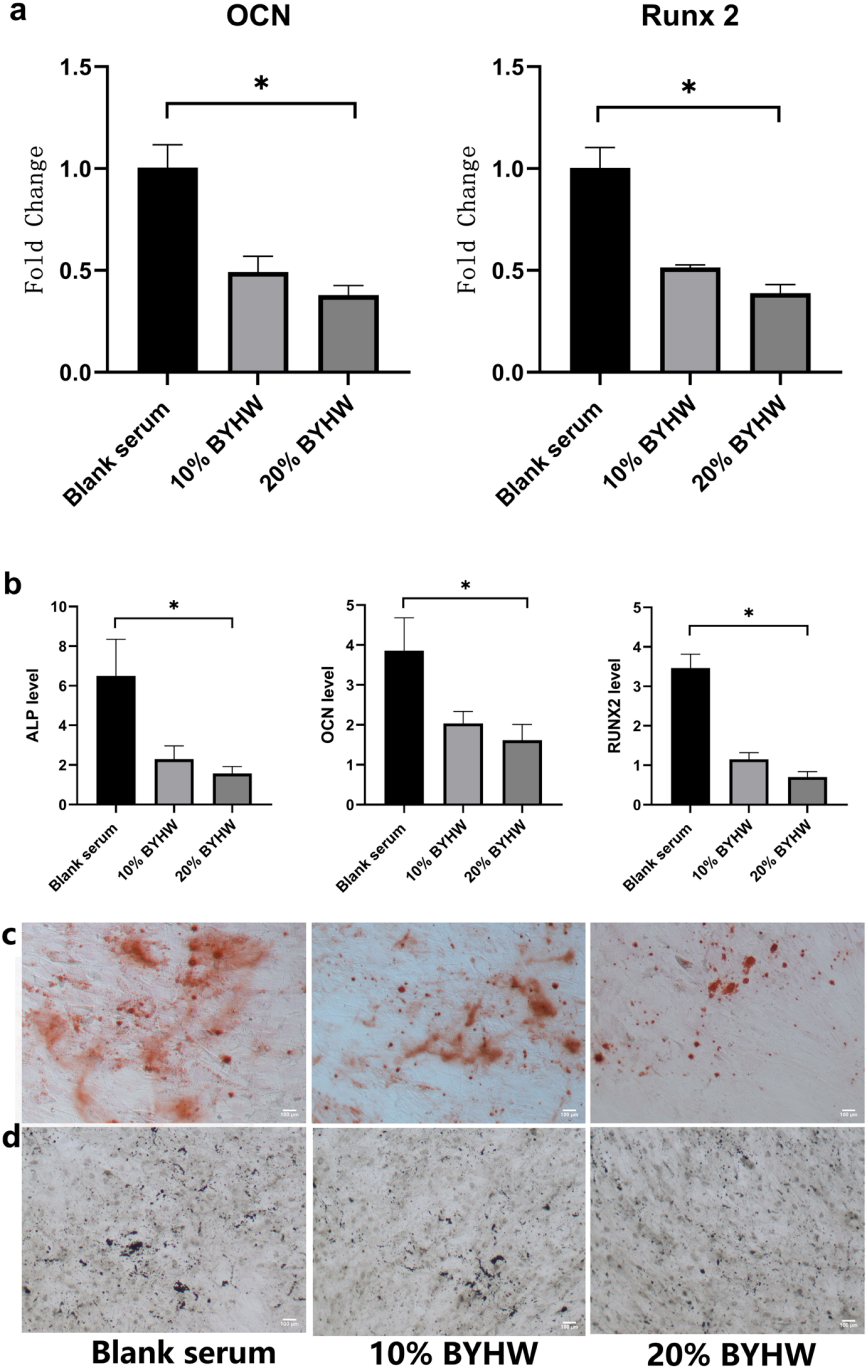
BYHW inhibited osteogenesis effect of MDSC. (a) PCR analysis of the expression profile of RUNX2 and OCN. (b) ELISA analysis of the expression profile of ALP, RUNX2 and OCN. (c) Alizarin Red staining demonstrating calcium deposition in different groups. (d) Immunohistochemistry showing the presence and distribution of ALP. * indicates *p* < 0.05. BYHW = Bu Yang Huan Wu. MDSC = Muscle‐derived stromal cells; PCR = polymerase chain reaction; ELISA = enzyme‐linked immunosorbent assay. ALP = alkaline phosphatase; RUNX2 = runtrelated transcription factor 2; OCN = osteocalcin.

The PCR and ELISA results confirmed that BYHW could significantly decrease the level of OSM, and high concentration (20% BYHW) was more effective than low concentration (10% BYHW) (Figure 3). In addition, compared with blank serum group, BYHW could restrain the level of osteogenic gene and protein (Figure 4a,b). Moreover, histology data revealed that calcium deposition as stained positive by Alizarin Red reduced obliviously in 10% and 20% BYHW group (Figure 4c). We also found that the positive staining of ALP weakened in BYHW group than that in blank serum group. Similarly, the effect in high concentration group (20% BYHW) was more significant. The results indicated that BYHW could inhibit the osteogenic differentiation of MDSC through restraining the OSM.

### 3.3 Interdicting JAK/STAT Signal In Vitro Suppressed the Influence of BYHW on MDSC Through OSM Mediated by Macrophages

**FIGURE 5:**
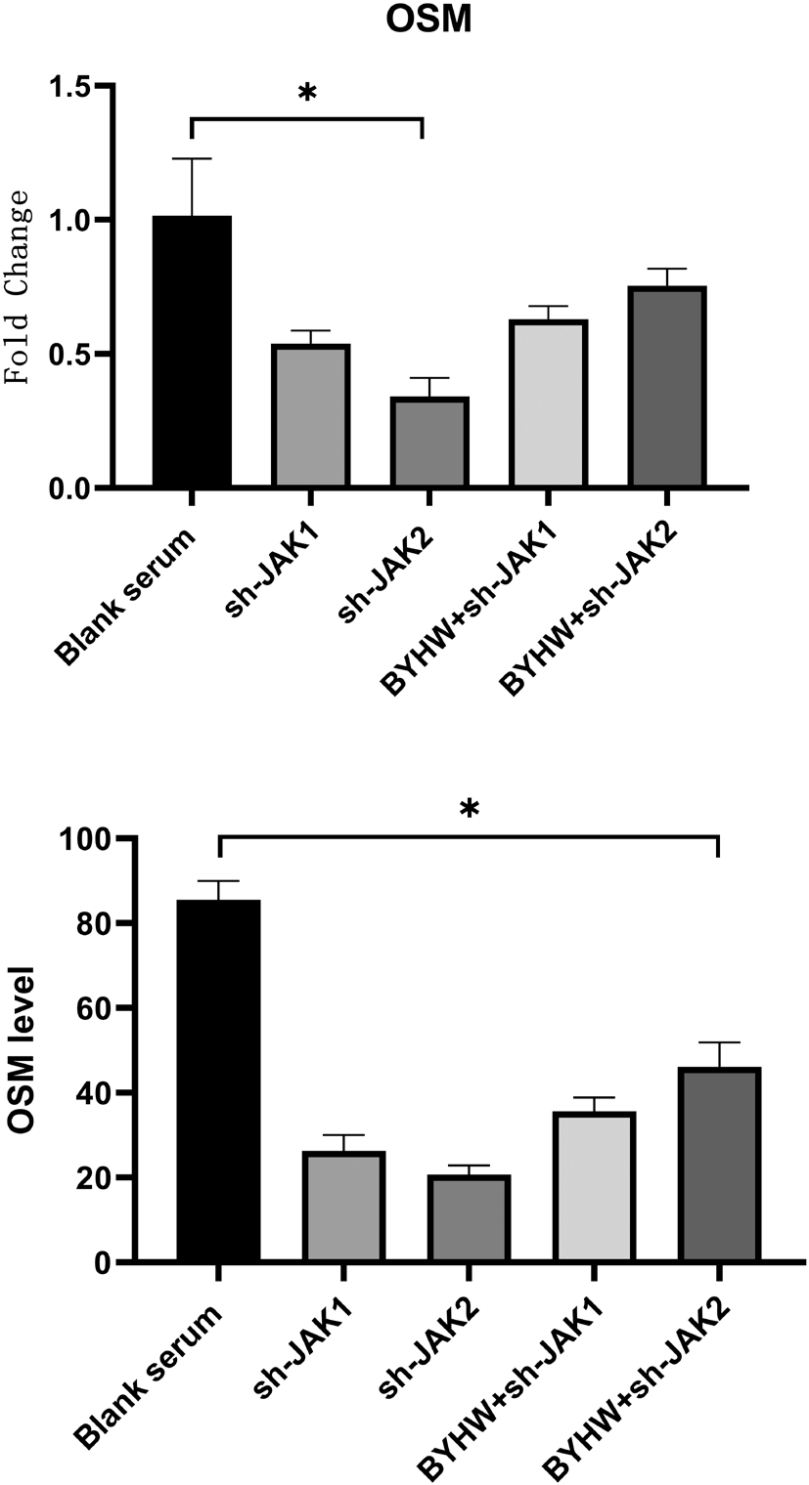
Interdicting JAK/STAT signal suppressed the level of OSM. OSM level measured by PCR and ELISA. * indicates *p* < 0.05. JAK/STAT = Janus kinase/signal transducer and activator of transcription. OSM = oncostatin M; PCR = polymerase chain reaction; ELISA = enzyme‐linked immunosorbent assay.

**FIGURE 6:**
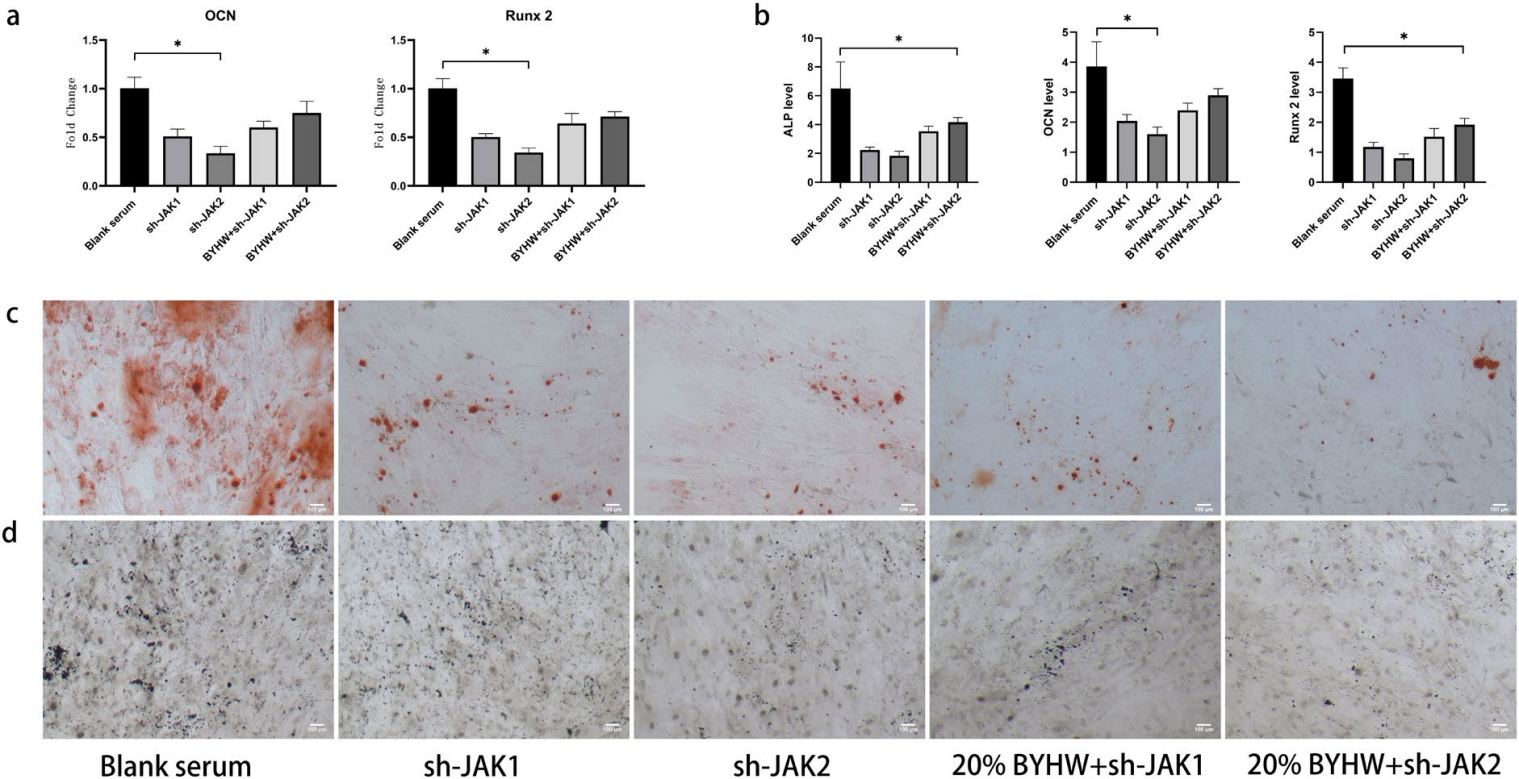
Interdicting JAK/STAT signal suppressed osteogenesis effect of MDSC. (a) PCR analysis of the expression profile of RUNX2 and OCN. (b) ELISA analysis of the expression profile of ALP, RUNX2 and OCN. (c) Alizarin Red staining demonstrating calcium deposition in different groups. (d) Immunohistochemistry showing the presence and distribution of ALP. * indicates *p* < 0.05. JAK/STAT = Janus kinase/signal transducer and activator of transcription; MDSC = Muscle‐derived stromal cells; PCR = polymerase chain reaction; ELISA = enzyme‐linked immunosorbent assay. ALP = alkaline phosphatase; RUNX2 = runtrelated transcription factor 2; OCN = osteocalcin.

To explore the effect of JAK/STAT signal on MDSC osteogenic activity through OSM mediated by macrophages, we firstly used lentiviruses to knock down JAK1 and JAK2, then added BYHW serum to make further evaluation. We found that the level of OSM markedly decreased in sh‐JAK1, sh‐JAK2, 20% BYHW+sh‐JAK1 and 20% BYHW+ sh‐JAK2 group compared with blank serum group. The level of OSM in sh‐JAK1 group, sh‐JAK2 group, 20% BYHW+sh‐JAK1 group and 20% BYHW+sh‐JAK2 showed no significant dereference (Figure 5). In addition, we found that the level of osteogenic gene and protein were downregulated in sh‐JAK1 group, sh‐JAK2 group, 20% BYHW+sh‐JAK1 group and 20% BYHW+ sh‐JAK2 group (Figure 6a,b). The Alizarin Red and immunohistochemistry also confirmed the attenuation of osteogenesis (Figure 6c).

**FIGURE 7:**
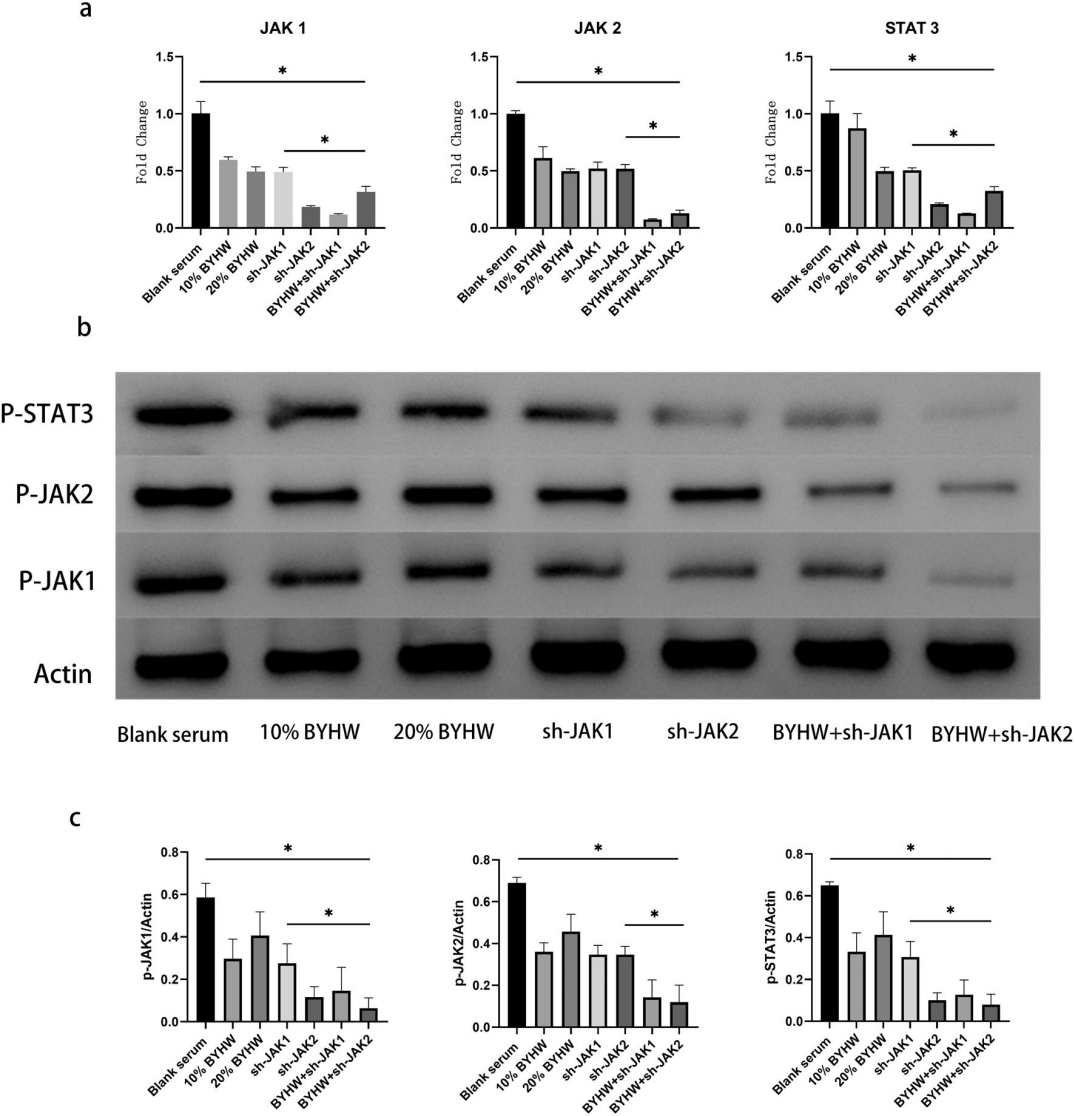
BYHW and lentivirus inhibited JAK/STAT signal. PCR and WB were performed to measure related gene and protein expression (JAK 1, JAK 2 and STAT 3) of JAK/STAT pathway. (a) PCR analysis of the expression profile of related gene in JAK/STAT signal;(b) WB analysis of the expression profile ofrelated protein in JAK/STAT signal;(c) WB gray level analysisof related protein in JAK/STAT signal. * indicates p < 0.05.

To explore the effect of BYHW on JAK/STAT pathway, we evaluated critical components of this pathway. The PCR and WB results indicated the level of related components (JAK1, JAK2 and STAT1) significantly reduced in BYHW or sh‐JAK group compared with blank serum group. In addition, the level of related components reached the lowest value when BYHW and sh‐JAK were combined (Figure 7). These results suggest that blocking of JAK/STAT pathway can suppress the level of OSM and osteogenic differentiation of MDSC. BYHW and lentiviruses can inhibit JAK/STAT pathway.

## 4 Discussion

HO is characterised by the ectopic bone formation in the soft tissue, involved with inflammation following trauma, aberrant stem cell recruitment and differentiation [22, 23]. Macrophage is an essential inflammatory cell, which can enhance the transition of quiescent mesenchymal stem cells (MSC), also stimulate MSC production from cycling MSC [22]. MDSC is the MSC in muscle with osteogenic differentiation potential [6, 7, 8]. Thus, osteogenic differentiation of MDSC mediated by macrophage is the main reason of HO formation. Previous studies have demonstrated BYHW could inhibit inflammation, improve microcycle and prevent HO [17, 24]. However, the exact molecular mechanism behind this phenomenon is still unclear. Recent evidence showed that OSM secreted by activated macrophage could promote osteogenic differentiation of MSC and the potential of BYHW in inhibiting osteogenic differentiation of MSC via JAK/STAT signalling‐dependent manner [1, 20, 25]. Therefore, we hypothesised that JAK/STAT signalling was integral to the mechanism of MDSC's osteogenic differentiation through OSM. We designed a shRNA to knock‐down JAK expression in MDSC, revealed that BYHW could inhibit osteogenic differentiation of MDSC mediated by OSM via suppressing JAK/STAT signalling. These findings provide evidence of a novel mechanism and a potential therapeutic target for acquired HO.

Many signalling pathways are known to be involved in MDSC aberrant osteogenic differentiation in HO formation. Of these, we focused on the JAK/STAT signalling pathway because of its importance in HO progression. As a member of the interleukin‐6 family, OSM could lead to the dimerisation of CD130 and activate JAK (JAK 1 and JAK 2); then, tyrosine phosphorylate downstream STAT to form homodimers or heterodimers; next, phosphorylated (p)‐STAT could enter the nucleus and regulate a series of biological process [26]. Nicolaidou et al. found that OSM produced by macrophages could activate STAT3 and drive MSC differentiation into osteoblasts. This study confirmed the local activation of STAT3 in MSC may be a valuable tool to increase the potential of MSC and HO formation [11]. Furthermore, Alexander and his colleagues demonstrated that using JAK inhibitor ruxolitinib blocked OSM‐driven STAT tyrosine phosphorylation in MDSC. Their in vivo research indicated administration of ruxolitinib significantly reduced STAT phosphorylation in injured muscles as well as the risk of HO [19]. Similarly, Yang et al. found recombinant OSM could induce osteogenic differentiation of ligamentum flavum cells and upregulate the expression of OSM receptor, CD130, p‐JAK2 and p‐STAT3. Upon knockdown of OSMR or CD130 could decrease p‐JAK2 and p‐STAT3 expression and osteogenic differentiation of ligamentum flavum cells [20]. A latest study showed that OSM could promote osteogenic differentiation of tendon‐derived stem cells and blocking JAK/STAT signalling decreased expression of pathway‐related proteins, as well as osteogenic genes [25]. Thus, inhibition of JAK/STAT signalling pathway could suppress the function of OSM secreted by macrophages and the osteogenic differentiation of MDSC, then interfere with the formation of acquired HO. JAK/STAT signalling pathway is a potential therapeutic target to inhibit osteogenic differentiation and reduce HO risk.

The pathogenesis of TCM for HO is summarised as qi stagnation and blood stasis, meridian obstruction, blood stasis accumulated in muscle tissue, and then mass induration formed over time. The BYHW decoction has long been used to treat stroke‐related diseases [27]. Based on the theory of TCM, BYHW has the effects of tonifying qi, invigorating blood, removing blood stasis and resolving hard mass. Basic researches have shown that BYHW could inhibit inflammation and macrophage activation, improve microcirculation, alleviate tissue damage caused by ischemia and hypoxia [24, 28]. What was more, it was reported that BYHW could inhibit JAK/STAT pathway [29, 30]. In addition, clinical studies have further confirmed that BYHW could reduce the risk of acquired HO [17]. However, the underlying molecular mechanism of BYHW on preventing osteogenic differentiation of MDSC and HO formation remains poorly understood. In this study, we found that BYHW could suppress the level of OSM and osteogenic differentiation of MDSC via inhibiting JAK/STAT pathway.

Many signalling molecules are involved in regulating the osteogenic differentiation of stem cells and HO formation. Our previous study demonstrated that the presence of muscle injury could cause a systemic decrease in circulating transforming growth factor‐beta1 (TGF‐β1), which promoted HO following muscle injury. We further showed that suppression of TGF‐β signalling could promote potentiated osteogenic differentiation of MDSC and HO formation [7]. Following research confirmed downregulation of TGF‐β1 in MDSC resulted in hyperinflammatory state, subsequent failed muscle repair and HO formation [31]. Zhang et al. isolated the osteoprogenitors from human HO samples resulting from the critical elbow trauma, showing that the activation of extracellular signal‐regulated kinase and hedgehog signalling pathway enhanced the osteogenic potential of osteoprogenitors to induce the formation of traumatic HO, when related pathways were inhibited, osteogenic potential of osteoprogenitors decreased significantly [32]. In addition, Kan et al. performed the signalling pathway analysis for immune‐MSC interaction molecular pairs using scRNA‐seq and spatial transcriptome database and found that genes associated with PI3K/AKT signalling pathway was enriched, suggesting that PI3K/AKT signalling regulated aberrant osteochondral differentiation of MSC during HO formation [22]. Our study showed that BYHW or lentivirus could inhibit JAK/STAT pathway; the application of BYHW and lentivirus jointly further reduces the expression of related factors, inhibiting JAK/STAT pathway.

There are some limitations to this study. First, we did not classify the type of macrophages. It was reported that M1 macrophages‐derived OSM induced osteogenic differentiation while M2 macrophages were found to promote chondrogenesis [22]. Second, animal experiment was missing. We have established an animal model of dystrophic calcification/HO through combination of hindlimb amputation and muscle injury (cardiotoxin) [33]. We would evaluate the effect of BYHW in preventing HO on animal, even clinical patients in subsequent research. The last but not least, OSM promoted osteogenic differentiation of stem cells via multiple signalling pathways [7, 22, 32]. We only investigated the JAK/STAT signalling pathway in this study. To address the above limitations, future research is necessary.

## 5 Conclusion

In conclusion, our results demonstrate that BYHW can inhibit osteogenic differentiation through OSM mediated by macrophages via suppressing the JAK/STAT signalling pathway in MDSC. Overall, these results suggest that JAK/STAT pathway may be a potent potential therapeutic target for abnormal osteogenic differentiation and HO formation.

## Author Contributions

**Guorui Cao:** writing – original draft (equal), writing – review and editing (equal). **Shaoyun Zhang:** writing – review and editing (equal). **Yuanping Liao:** investigation (equal), methodology (equal). **Chen Yue:** data curation (equal), formal analysis (equal). **Lanbo Yang:** resources (equal), software (equal). **Jiayi Guo:** validation (equal), visualization (equal). **Peijian Tong:** supervision (equal). **Honglue Tan:** project administration (equal), supervision (equal).

## Consent

The patients consented to the femoral heads samples being taken for the purpose of research and also consented to their publication.

## Conflicts of Interest

The authors declare no conflicts of interest.

## Data Availability

The datasets used and/or analysed during the current study are available from the corresponding author on reasonable request.
